# Biodegradable Double-Layer Hydrogels with Sequential Drug Release for Multi-Phase Collaborative Regulation in Scar-Free Wound Healing

**DOI:** 10.3390/jfb16050164

**Published:** 2025-05-07

**Authors:** Xinyu Zhang, Qianhe Zu, Chunlin Deng, Xin Gao, Hongxu Liu, Yi Jin, Xinjian Yang, Enjun Wang

**Affiliations:** 1 College of Nursing, Hebei University, Baoding 071002, China; 20228025012@stumail.hbu.edu.cn (X.Z.); 20238025011@stumail.hbu.edu.cn (H.L.); 2 College of Basic Medical Science, Key Laboratory of Pathogenesis Mechanism and Control of Inflammatory-Autoimmune Diseases of Hebei Province, Hebei University, Baoding 071002, China; 20222101002@stumail.hbu.edu.cn (Q.Z.); jinyi2009@hbu.edu.cn (Y.J.); 3 College of Chemistry & Materials Science, State Key Laboratory of New Pharmaceutical Preparations and Excipients, Key Laboratory of Medicinal Chemistry and Molecular Diagnosis of the Ministry of Education, Chemical Biology Key Laboratory of Hebei Province, Institute of Life Science and Green Development, Hebei University, Baoding 071002, China; 20227016056@stumail.hbu.edu.cn (C.D.); gx2025@stumail.hbu.edu.cn (X.G.)

**Keywords:** double-layer hydrogels, sequential drug release, multi-phase regulation, scar-free wound healing

## Abstract

Scarring is a prevalent and often undesirable outcome of the wound healing process, impacting millions worldwide. The complex and dynamic nature of wound healing, including hemostasis, inflammation, proliferation, and remodeling, necessitates precise, making it hard for stage-specific interventions to prevent pathological scarring. This study introduces a double-layer hydrogel system designed for sequential drug release, aligning with the stage-specific need for wound healing. The lower layer, containing curcumin-loaded chitosan nanoparticles, shows early anti-inflammatory and antioxidant effects, while the upper layer, with pirfenidone-encapsulated gelatin microspheres, presents late-stage anti-fibrotic activity. The hydrogel’s unique design, with varying degradation rates and mechanical properties in each layer, facilitates cascade drug release in synchrony with wound healing stages. Rapid release of curcumin from the lower layer promotes proliferation by mitigating inflammation and oxidative stress, while the sustained release of pirfenidone from the upper layer inhibits excessive fibrillation during late proliferation and remodeling. In a rat model of full-thickness skin defect, treatment with a double-layer hydrogel drug delivery system accelerated the wound closure, improved scar quality, and promoted the formation of hair follicles. Therefore, this innovative approach lays a promising foundation for future clinical applications in anti-scar therapies, offering a significant advancement in wound care and regenerative medicine.

## 1. Introduction

Scarring is a natural, but often undesirable, consequence of the wound healing process following surgery, trauma, burns, or acne-related skin injuries [1]. Millions of people worldwide annually suffer from pathological scars [2,3], which not only impact the appearance but also skin function, restricting mobility and causing psychological distress [4,5]. Despite numerous treatment options, including surgical intervention, laser therapy, pressure garments, and massage, have been applied for preventing scar formation, they have several limitations, such as secondary injuries from surgery, prolonged use of pressure garments, and risks like erythema and purpura for laser treatments [6,7]. Thus, developing safe, effective strategies for promoting rapid wound healing and preventing scar formation remains a critical clinical challenge.

Wound healing is a complex, multi-phase process involving hemostasis, inflammation, proliferation, and remodeling. Each phase holds a specific biological function, collectively restoring tissue homeostasis [8]. In the hemostasis process, platelets are activated immediately, triggering a coagulation cascade that rapidly seals the exposed tissues to prevent microbial invasion [9]. During inflammation, immune cells, including macrophages and neutrophils, will clear the pathogens and debris in the wound. Prolonged inflammation can lead to oxidative stress, which further deteriorates the tissue damage [10]. In the proliferation phase, fibroblasts and myofibroblasts secrete various extracellular matrix (ECM) proteins that are essential for wound closure. However, excessive fibroblast activation leads to abnormal collagen deposition and scarring [8]. The myofibroblasts drive wound contraction, but their overactivity can cause fibrosis. In the remodeling process, the ECM components are realigned, and their imbalance in this phase will result in permanent scarring [11]. This cascade wound healing process highlights the necessity for precise, stage-specific interventions to mitigate scar formation and facilitate scar-free wound healing.

Recent advance in biomaterials offers a promising avenue for addressing the challenge of scar-free wound healing. Among these biomaterials, hydrogels have gained much attention due to their excellent biocompatibility, biodegradability, and tunable properties. Hydrogels can serve as delivery systems for bioactive agents, enabling controlled and sustained release of drugs, growth factors, and stem cell-derived vesicles [12,13]. For instance, studies have demonstrated the potential of hydrogels loaded with mesenchymal stem cells (MSCs), transforming growth factor-β (TGF-β), or extracellular vesicles to reduce inflammation and promote tissue regeneration [14,15,16,17]. However, these approaches are fundamentally limited by their inability to achieve precise spatiotemporal control over therapeutic delivery—a critical requirement for phase-specific targeting in wound healing. Multi-layer hydrogel systems address this challenge through architecturally programmed sequential drug release mechanisms, which synchronize therapeutic actions with the dynamic biological progression of tissue repair [18]. Compared to conventional single-layer hydrogels, the rationally designed double-layer system enables simultaneous administration of multiple therapeutics with temporally distinct release profiles, thereby achieving comprehensive regulation across healing phases. Consequently, the precisely engineered double-layer hydrogel facilitates coordinated intervention during critical inflammatory and proliferative windows, effectively preventing pathological scarring through dual suppression of excessive immune responses and ECM accumulation.

**Scheme 1:**
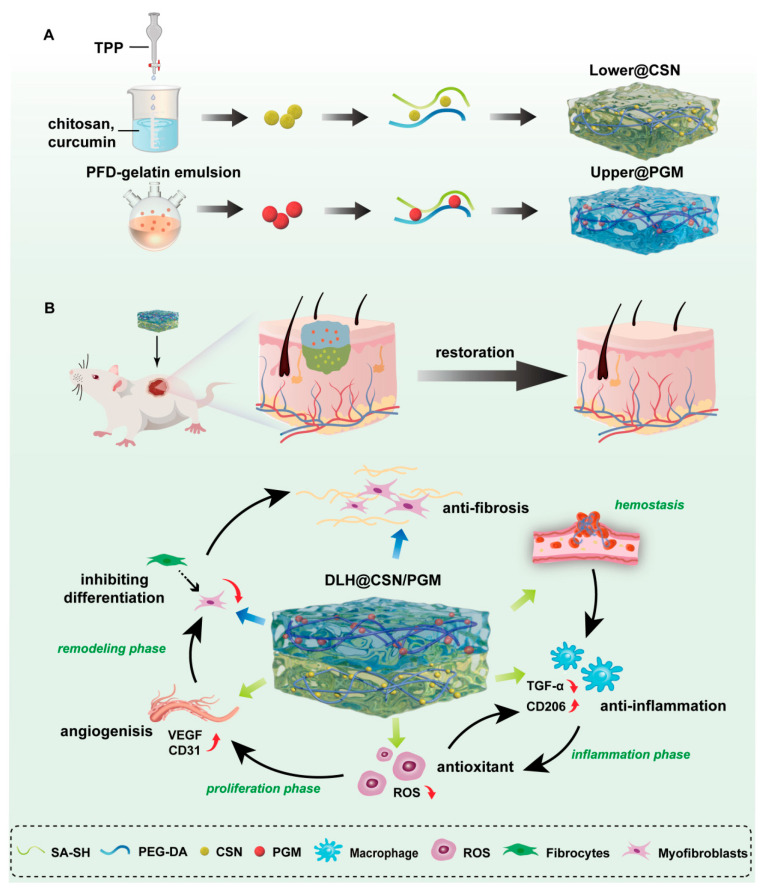
Schematic illustration. (**A**) The synthesis of the double-layer hydrogel. (**B**) The double-layer hydrogel drug delivery system with sequentially released drugs promotes scar-free wound healing through multi-stage collaborative regulation.

In this study, we propose a novel double-layer hydrogel drug delivery system (DLH@CSN/PGM) for sequential drug release to interfere with each wound healing phase. The lower hydrogel layer is crosslinked with thiolated alginate (SA-SH) and low-molecular-weight polyethylene glycol diacrylate (PEG-DA, 400 Da). In contrast, the upper layer is crosslinked using high-molecular-weight PEG-DA (700 Da), forming a denser and slower-degrading matrix. This molecular weight-engineered stratification ensures distinct mechanical properties and degradation profiles between the two layers. Chitosan nanoparticles were selected to encapsulate curcumin (Cur) for enhanced bioavailability and reactive oxygen species (ROS) scavenging [19,20], while gelatin microspheres (GEMs) enabled matrix metalloproteinase (MMP)-responsive pirfenidone (PFD) release [21,22,23]. Unlike conventional bilayer systems relying on homogeneous crosslinkers, the molecular weight-modulated PEG-DA architecture creates a degradation gradient aligned with healing phases—a critical improvement enabling scar prevention through stage-matched drug kinetics. This strategic configuration facilitates precise spatiotemporal control of drug release, with Cur loaded in the lower layer (Lower@CSN) for early anti-inflammatory and antioxidant effects [24,25] and PFD incorporated into the upper layer (Upper@PGM) for late-stage anti-fibrotic activity [26,27]. By synchronizing the release of these drugs, this hydrogel system aims to systematically regulate inflammation, promote ECM deposition during the early stages, and prevent fibroblast overactivation and excessive collagen synthesis in the later stages (Scheme 1). This integrated approach is expected to optimize the wound healing process, facilitating rapid and scar-free recovery.

## 2. Materials and Methods

### 2.1. Preparation and Characterization of (DLH@CSN/PGM)

#### 2.1.1. Lower@CSN Hydrogel

The Lower@CSN was prepared by dissolving 10 mg of photoinitiator I2959 in 5 mL deionized water, followed by adding 100 μL of PEG-DA (Mn = 400) and 1.5 mg/mL of Cur -loaded chitosan nanoparticles (CSNs). After thorough stirring, the solution was combined with 0.075 g of SA-SH, stirred rapidly for 10 min, and irradiated under an ultraviolet (UV) lamp. The final materials were then dialyzed against deionized water for 24 h using a dialysis bag with a molecular weight cutoff of 14 kDa to remove unreacted I2959 and PEG-DA [16].

#### 2.1.2. Upper@PGM Hydrogel

For the Upper@PGM, 10 mg of I2959 was dissolved in 5 mL deionized water, followed by adding 165 μL of PEG-DA (Mn = 700) and 1 mg/mL of PFD-encapsulated gelatin microspheres (PGMs). Then, the solution was mixed with 0.075 g SA-SH, stirred, and irradiated under UV for 10 min. The final materials were dialyzed against deionized water for 24 h using a dialysis bag with a molecular weight cutoff of 14 kDa to eliminate unreacted components.

#### 2.1.3. Characterization

To investigate the morphology of the hydrogels, samples were examined on a scanning electron microscope (SEM) operated at 15 kV. The functional groups of the fabricated scaffolds were analyzed using Raman spectroscopy and Fourier transform infrared spectroscopy (FTIR).

### 2.2. In Vitro Drug Release Study

The DLH@CSN/PGM were incubated in 10 mL of phosphate-buffered saline (PBS) at pH values (4.5, 7.4, and 8.5) with continuous shaking (37 °C). Samples were collected at predefined intervals to capture early burst release and sustained phases. At each time point, 1 mL of the release medium was withdrawn and replaced with an equal volume of fresh PBS to maintain a constant volume. Cur and PFD concentrations were quantified using ultraviolet–visible (UV–Vis) spectroscopy at 425 nm and 281 nm, regarding pre-established calibration curves (Figures S10 and S11). The calculation formula of drug released (%) is as follows:

(1) $$Drug\;released\;(\%)=\frac{M_{t}}{M_{\infty }}\times 100$$

where $M_{t}$ and $M_{\infty }$ are the mass of the drug released at time t and the total mass of the hydrogel loaded with the drug, respectively.

### 2.3. Cell Proliferation and Migration Assay

Cell proliferation was assessed using a 5-Ethynyl-2′-deoxyuridine (EdU) Cell Proliferation Kit with Alexa Fluor 555 (Meilun, Dalian, China) following the manufacturer’s protocol. Cell migration was evaluated via scratch and transwell assays. In the scratch assay, National Institutes of Health (NIH)-3T3 cells were cultured to 80% confluence in 6-well plates, scratched with a pipette tip, rinsed with PBS, and treated with Dulbecco’s modified Eagle medium (DMEM) containing DLH@CSN/PGM extracts for 24 or 48 h. Images were captured using an inverted fluorescence microscope. For the transwell assay, cells in the upper chamber migrated toward DLH@CSN/PGM-containing medium in the lower chamber. After 24 or 48 h, migratory cells were fixed, stained, and imaged.

### 2.4. Tube Formation Assay

The effect of hydrogel on angiogenesis was detected by tube formation assay. Matrigel (50 μL/well) was added to a pre-chilled 96-well plate and solidified at 37 °C. Human umbilical vein endothelial cell (HUVEC) suspensions were seeded onto the gel and incubated for 6 h at 37 °C. Tube formation was imaged and quantitatively analyzed using ImageJ software (version 1.54f).

### 2.5. Antioxidant Activity

The antioxidant activity of DLH@CSN/PGM was assessed using a 1,1-diphenyl-2-picrylhydrazyl radical 2,2-diphenyl-1-(2,4,6-trinitrophenyl)ydrazyl (DPPH) free radical scavenging assay. The DLH@CSN/PGM extract was mixed with DPPH ethanol solution and incubated in the dark for 30 min. The absorbance of the solution was measured using a UV–Vis spectrophotometer. The DPPH scavenging rate was calculated as follows:

(2) $$DPPH\;scavenging\;(\%)=\frac{A_{c}-A_{s}}{A_{c}}\times 100$$

where A_c_ and A_s_ are the absorbances of the Control and sample, respectively.

For intracellular ROS scavenging, NIH-3T3 cells were pretreated with Rosup and incubated with hydrogel extract or fresh medium for 24 h. The cells were then stained with dichlorofluorescein diacetate (DCFH-DA) and observed on a fluorescence microscope.

### 2.6. In Vivo Wound Healing

Eight-week-old female Sprague–Dawley (SD) rats were used to establish a full-thickness skin defect model. All animal experiments were conducted in accordance with the guidelines set by the Animal Welfare and Ethical Committee of Hebei University (No. IACUC-2021XS027). The experimental groups (six rats per group) received different hydrogel treatments (Control, double-layer hydrogels (DLHs), Lower@CSN, Upper@PGM, and DLH@CSN/PGM), while the Control group was treated with a 3M transparent adhesive patch. Wound healing was monitored on days 0, 3, 7, and 12 post-wounding, with wound areas photographed and analyzed using ImageJ software. Scar length was measured on day 12.

(3) $$Relative\;Wound\;Area\;(\%)=\frac{\left(S_{0}-S_{t}\right)}{S_{0}}\times 100$$

where S_0_ and S_t_ represent the initial wound area and the wound area at different time points, respectively.

### 2.7. Histological Evaluation

Trauma tissue samples were either fixed in 4% paraformaldehyde or rapidly frozen. After fixation, the samples were paraffin-embedded and sectioned for subsequent staining. Histological evaluations included hematoxylin and eosin (H&E) staining, Masson’s trichrome staining, Sirius Red staining, immunohistochemical (IHC) staining, immunofluorescence staining, and dihydroethidium (DHE) immunofluorescence staining.

### 2.8. Statistical Analysis

All experimental data, obtained from at least three independent experiments, were analyzed using GraphPad Prism 8 software. Results are presented as differences among multiple groups, with pairwise comparisons between two groups performed using independent samples *t*-tests. *p* < 0.05 was considered statistically significant.

## 3. Results and Discussion

### 3.1. Synthesis and Characterization of DLH@CSN/PGM

**Figure 1:**
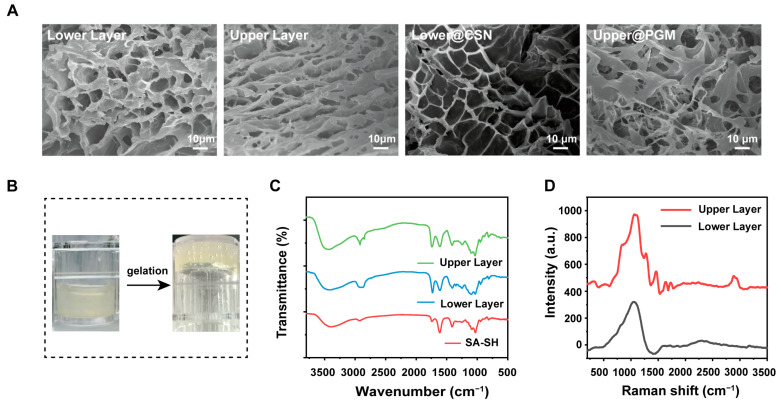
Synthesis and characterization of DLH@CSN/PGM. (**A**) SEM of DLHs and DLH@CSN/PGM. (**B**) Photographs of DLHs. (**C**) FTIR spectra of SA-SH and DLH. (**D**) Raman spectra of DLHs.

The double-layer hydrogel was successfully synthesized using a crosslinking strategy that resulted in distinct upper and lower layers [16], as visually evidenced by the photographic images (Figure 1B and Figure S12). The FTIR spectra confirmed the chemical crosslinking of SA-SH and PEG-DA via thiol-ene click chemistry. Peaks observed at 1750 cm^−1^ (C=O) and 1300 cm^−1^ (R-O-R) belonging to PEG-DA in both layers validated the successful formation of the hydrogel network (Figure 1C). Raman spectroscopy (Figure 1D) confirmed the absence of a peak near 524 cm^−1^, which indicated that there were no disulfide bonds. The results of FTIR and Raman spectra jointly confirm that thiolated sodium alginate and PEG-DA were crosslinked through thiol-ene click chemical bonds rather than disulfide bonds. The thiol-ene click reaction between SA-SH and PEG-DA forms a crosslinked network via thiol (-SH) and acrylate group addition, exemplifying rapid, selective “click chemistry” for controlled hydrogel synthesis. SEM images revealed that both hydrogel layers were porous, and the upper layer was relatively dense, a critical feature for drug loading and release (Figure 1A).

To realize controlled drug release, Cur was encapsulated within chitosan nanoparticles using ionic gelation [28]. FTIR analysis confirmed the successful encapsulation of Cur, with characteristic peaks corresponding to the aromatic structure of Cur (1600~1500 cm^−1^) and functional groups of chitosan (-OH and -NH_2_) at 3400 cm^−1^ and 1600 cm^−1^, respectively (Figure S1). Transmission electron microscope (TEM) images revealed spherical nanoparticles with a particle size ranging from 100 to 200 nm (Figure S3), consistent with SEM findings. The drug loading rate of CSNs was about 21.4%. PFD was loaded into gelatin microspheres via emulsion crosslinking [29,30]. The FTIR spectra showed that the peak at 2922 cm^−1^ of GEMs and PGMs contributed to C=N caused by the successful crosslinking between gelatin and glutaral. The nearby peaks at 3100~3000 cm^−1^ for PFD and PGMs were due to the pyridine characteristic absorption peak (C-H). These results indicated that gelatin microspheres loaded PFD were successfully compounded (Figure S2). SEM images confirmed that gelatin microspheres had a uniform spherical morphology (~5 μm), which was suitable for controlled drug release (Figure S4). The drug loading rate of PGMs was about 11.25%. SEM analysis of DLHs containing CSNs and PGMs showed a loose and porous structure similar to the original DLH (Figure 1A).

### 3.2. Mechanical Properties and Swelling Behavior

**Figure 2:**
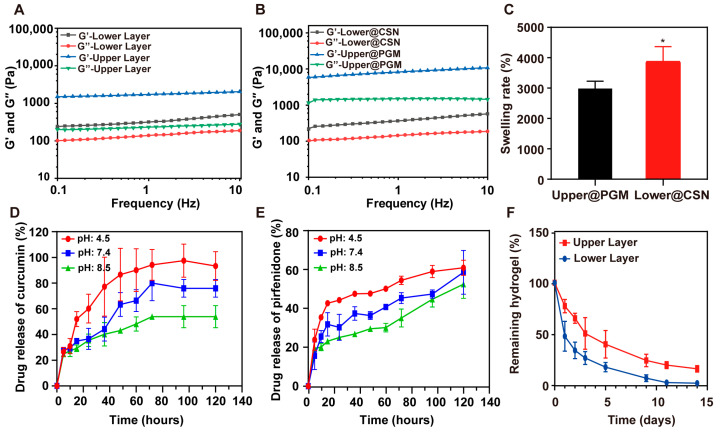
The mechanical properties, swelling, degradation, and drug release of DLH@CSN/PGM. (**A**,**B**) Mechanical properties of DLHs and DLH@CSN/PGM via frequency scan. (**C**) The swelling ratio of Upper@PGM and Lower@CSN (*n* = 3). * *p* < 0.05 vs. Upper@PGM. (**D**,**E**) Drug release at different pHs (*n* = 3). (**F**) Degradation behaviors of DLH@CSN/PGM (*n* = 3).

To test the robust mechanical performance of wound dressing application, rheological frequency tests were conducted on DLHs. For both layers, the storage modulus (G′) exceeded the loss modulus (G″), indicating excellent elastic stability (Figure 2A). This property is essential for maintaining structural integrity under physiological conditions. Similarly, the rheological profiles of Lower@CSN and Upper@PGM hydrogels displayed a consistent dominance of G′ over G″, further confirming their mechanical robustness and that the loading of CSNs and PGMs did not affect the mechanical robustness of hydrogels (Figure 2B). The swelling experiments revealed that the lower layer exhibited a significantly higher swelling rate than the upper layer (Figure 2C and Figure S5). This difference in swelling behavior can be attributed to the distinct hydrophilic properties of each layer. The lower layer, with its higher hydrophilicity, facilitates rapid water absorption, which is beneficial for initial wound hydration and drug diffusion. Such a difference was advantageous for spatiotemporal drug release with rapid drug diffusion in the lower layer and sustained release in the upper layer. Moreover, the excellent swelling capacity of hydrogel is conducive to the effective absorption of wound exudates.

### 3.3. Degradation Profiles and Sequential Drug Release

The degradation behavior of the DLH was assessed in PBS to simulate in vivo conditions. The lower hydrogel layer degraded more rapidly, with approximately 75% degradation on day 3, while the upper layer retained around 50% of its mass at the same time point (Figure 2F). This differential degradation rate is a critical design feature, as it ensures that the lower layer degrades quickly to release Cur for early anti-inflammatory effects, while the upper layer provides sustained release of PFD for late-stage anti-fibrotic action. This differential degradation aligned with the design intent, enabling sequential drug release to match the healing phases. Drug release studies in vitro revealed distinct release profiles for Cur and PFD under various pH conditions. Cur displayed a burst release in the lower layer, with ~50% released within the first day across all pH levels (4.5, 7.4, 8.5), reflecting its role in early-stage anti-inflammatory therapy (Figure 2D). In contrast, PFD showed a sustained release profile, with a slower drug release rate in the upper layer (Figure 2E). This phenomenon may be due to the fact that the upper hydrogel layer has a more compact structure than the lower hydrogel layer and the slow-release ability of GEMs. This double-release behavior ensured that the hydrogel could meet the spatiotemporal requirements of wound healing by providing early anti-inflammatory and late-stage anti-fibrotic effects. The above results showed that the DLH@CSN/PGM system exhibited a sophisticated design enabling sequential drug release tailored to the whole wound healing phases. Its distinct physicochemical properties, mechanical stability, and degradation profiles supported early anti-inflammatory effects via Cur’s burst release and sustained anti-fibrotic action of PFD. This innovative system offers sequential drug delivery, effectively addressing early inflammation and late-stage fibrosis, demonstrating significant potential to promote scar-free wound healing.

### 3.4. Biocompatibility and Hemostatic Ability of the Hydrogels

**Figure 3:**
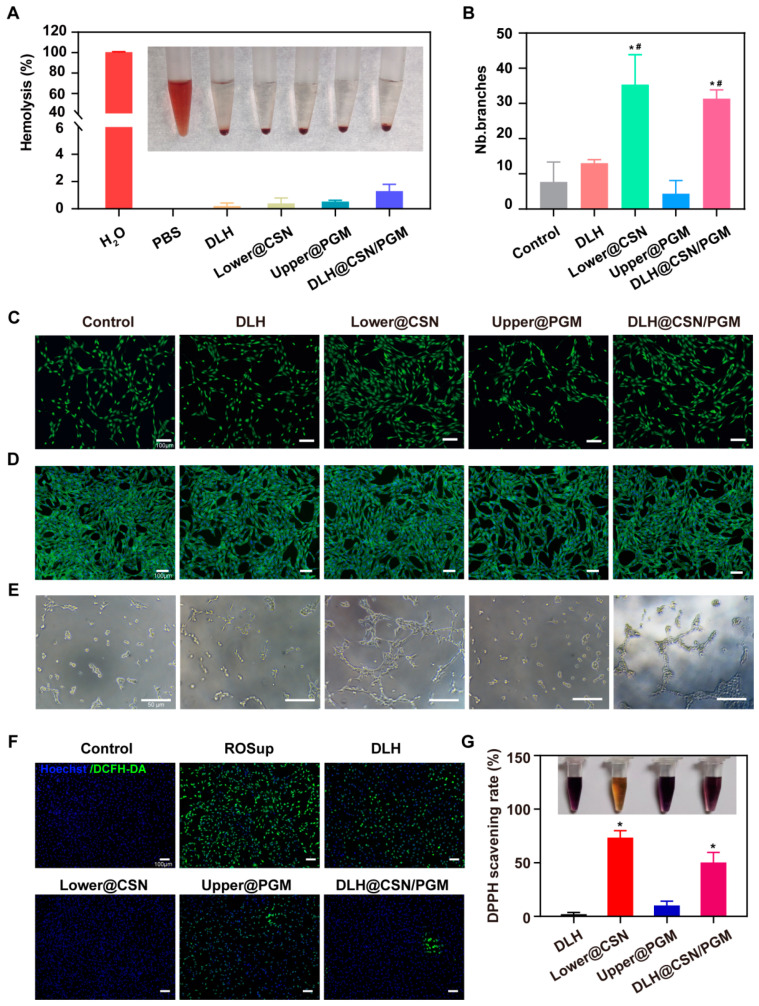
Evaluation of biocompatibility, angiogenesis, and antioxidant properties of hydrogels. (**A**) Hemolytic ratios of hydrogels. (**B**) Quantitative analysis of in vitro angiogenesis (*n* = 3). * *p* < 0.05 vs. Control; # *p* < 0.05 vs. DLH. (**C**) Live/Dead staining of NIH-3T3 cells, where green fluorescence from Calcein-AM indicates live cells and red fluorescence from propidium iodide (PI) indicates dead cells, bar: 100 μm. (**D**) Actin cytoskeleton staining of NIH-3T3 cells, bar: 100 μm. (**E**) In vitro angiogenic behavior of HUVECs, bar: 50 μm. (**F**) Fluorescence images showing intracellular ROS levels in NIH-3T3 cells after treatment with different hydrogels, with DCFH-DA staining, bar: 100 μm. (**G**) DPPH free radical scavenging assay (*n* = 3). * *p* < 0.05 vs. DLH.

The biocompatibility of this DLH@CSN/PGM is a critical factor for its application in wound healing. The blood compatibility was assessed through a hemolysis experiment, which is often used to evaluate the potential toxic effect of different materials to cause lysis of red blood cells. As shown in Figure 3A, the data indicated that all hydrogel formulations, including DLH@CSN/PGM, exhibited a hemolysis rate of less than 2%, which was well below the safety threshold of 5% for biological materials. This result suggests that the hydrogels are non-toxic and suitable for biological applications. To further evaluate the biocompatibility of the hydrogels, cell counting kit-8 (CCK-8) assay, Live/Dead staining, and cytoskeletal staining were conducted on NIH-3T3 cells. The CCK-8 assay result demonstrated that the cell viability remains above 80% for all treatment groups at both 24 and 48 h, with a significant increase in the Lower@CSN and DLH@CSN/PGM groups at 48 h (Figure S6). Live/Dead staining results revealed that there are minimal dead cells, indicated by red fluorescence across all treatment groups (Figure 3C), suggesting the cell survival supporting effect of the hydrogels. Actin cytoskeleton staining result showed well-maintained cellular morphology in Control and treated groups (Figure 3D), further confirming the cytocompatibility of the hydrogel system.

Effective hemostasis is essential for wound dressings, particularly in the immediate aftermath of injury. In vivo experiments using a liver hemorrhage model (Figure S7) demonstrated that the DLH@CSN/PGM hydrogel significantly reduced blood loss compared to the Control group. This was quantified by a substantial decrease in blood loss, indicating the hydrogel’s potential for effective hemostasis in wound management. Therefore, the double-layer hydrogel system demonstrates excellent biocompatibility and hemostatic properties, which are essential for its potential wound healing applications.

### 3.5. In Vitro Antioxidant Capacity

ROS have both beneficial and detrimental effects in wound healing depending on their concentration. ROS with low concentration defend against pathogens and promote the healing of the wound, while excessive ROS will lead to oxidative stress, cell damage, and impairment of the wound healing process [31]. To assess the antioxidant capacity of this hydrogel system, the DPPH assay and DCFH-DA staining experiments were conducted. The DPPH assay results revealed significant differences in free radical scavenging activity among the hydrogel formulations (Figure 3G). Specifically, DLH, Lower@CSN, Upper@PGM, and DLH@CSN/PGM exhibited DPPH radical scavenging activities of 2.09%, 73.48%, 10.18%, and 50.29%, respectively. The pronounced antioxidant activity of the Lower@CSN and DLH@CSN/PGM hydrogels can be attributed to the presence of Cur, which is known for its potent antioxidant properties. Complementary to the DPPH assay, DCFH-DA staining was used to visualize the intracellular ROS in NIH-3T3 cells (Figure 3F). The fluorescence images demonstrated a marked increase in green fluorescence in cells stimulated with ROS, indicative of high ROS levels. However, the treatment with Lower@CSN and DLH@CSN/PGM resulted in a noticeable reduction in green fluorescence intensity, confirming the effective ROS removal by these hydrogel formulations. The antioxidant properties of the hydrogels are crucial for the early stages of wound healing, where they can mitigate oxidative stress and protect cells from damage. The Lower@CSN and DLH@CSN/PGM, with their superior antioxidant capacities, are expected to provide an early anti-inflammatory and antioxidant effect, setting the stage for a controlled wound healing process.

### 3.6. In Vitro Angiogenesis Capacity

Angiogenesis is a critical process during the proliferative phase of wound healing, providing oxygen and nutrients necessary for tissue regeneration. However, uncontrolled angiogenesis leads to excessive scar formation [32,33]. DLH@CSN/PGM is designed to modulate angiogenesis by releasing Cur from the lower layer, which has been shown to promote angiogenesis in non-cancerous tissues. Cur enhances the secretion of vascular endothelial growth factor (VEGF) in endothelial progenitor cells (EPCs), thereby promoting cell proliferation and wound healing through the control of inflammation and oxidative stress [34,35]. The upper layer, containing PFD, is intended to inhibit excessive cell proliferation and prevent the over-deposition of the ECM by down-regulating TGF-β [36], thus preventing late-stage fibrosis and scarring.

The in vitro angiogenesis assay results demonstrated a significant increase in tubule formation in the Lower@CSN and DLH@CSN/PGM groups compared to the Control, DLH, and Upper@PGM groups (Figure 3E). The quantitative analysis of the number of branches (Figure 3B) showed that the Control, DLH, Lower@CSN, Upper@PGM, and DLH@CSN/PGM groups had an average of 7.67, 13.00, 35.33, 4.33, and 31.33 branches, respectively. The enhanced angiogenic effect of the Lower@CSN and DLH@CSN/PGM was attributed to the controlled release of Cur, which would stimulate the formation of new blood vessels without causing over-proliferation or excessive ECM deposition. The controlled release of Cur during the early stages of wound healing was expected to facilitate angiogenesis without leading to excessive scarring, as the later stages would be modulated by the release of PFD from the upper layer, which could inhibit the overactive angiogenesis and fibrosis.

### 3.7. In Vitro Cell Proliferation and Migration

**Figure 4:**
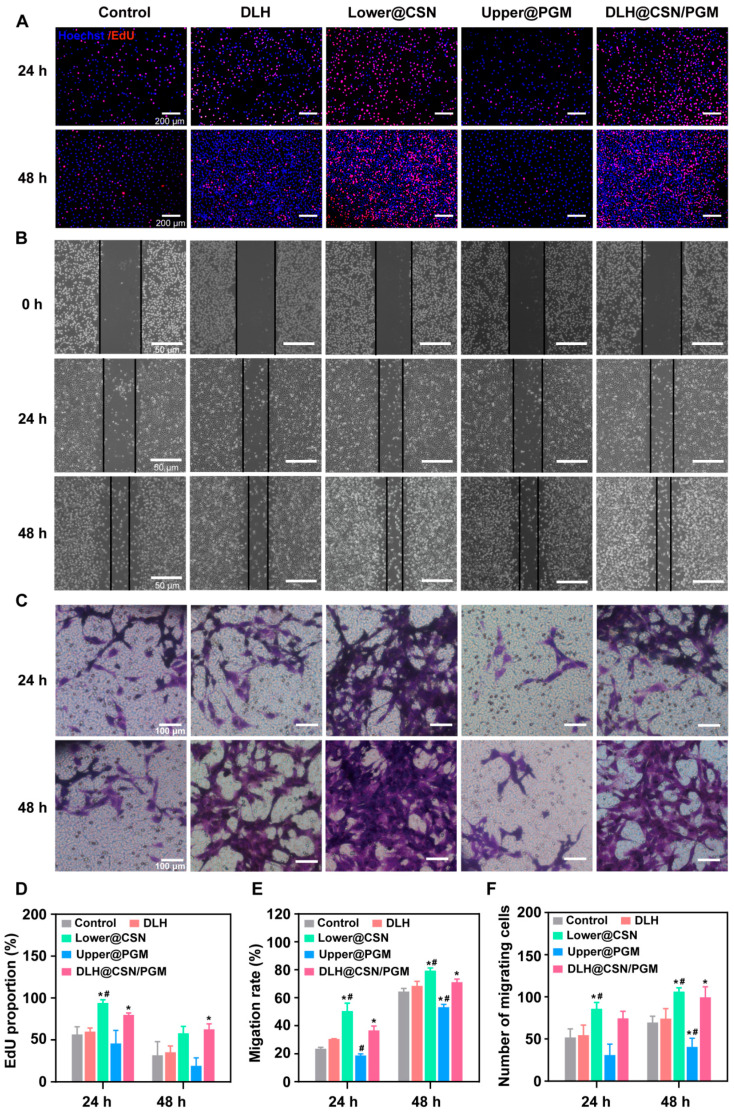
Assessment of the promotional effects on NIH-3T3 cell proliferation and migration by various hydrogels. (**A**) Fluorescence images depicting EdU staining to measure cell proliferation at 24 h and 48 h, bar: 100 μm. (**B**) Representative images from the cell scratch assay at 24 h and 48 h, bar: 50 μm. (**C**) Images from the Transwell migration assay at 24 h and 48 h, bar: 100 μm. (**D**) Quantitative analysis of EdU-positive cells as a proportion of total cells (*n* = 3). (**E**) Quantitative analysis of the migration rate in the scratch assay (*n* = 3). (**F**) Quantitative analysis of the number of migrating cells in the Transwell assay (*n* = 3), * *p* < 0.05 vs. Control; # *p* < 0.05 vs. DLH.

An effective wound healing strategy requires a delicate balance between promoting cell proliferation and migration during the initial stages and preventing excessive cellular activity that could lead to scarring [37]. DLH@CSN/PGM is engineered to keep this balance through the sequential release of bioactive molecules tailored to the different phases of wound healing. To assess cell proliferation, we used the EdU cell proliferation kit, which stains proliferating cells with red and nuclei with blue. As shown in Figure 4A, the degree of cell proliferation was comparable between the Control and DLH groups, and the Lower@CSN and DLH@CSN/PGM groups exhibited the highest number of EdU-positive cells at both 24 and 48 h, indicating robust cell proliferation. In contrast, the Upper@PGM group showed a significantly lower number of proliferating cells due to the anti-proliferative effect of PFD. Quantitative analysis confirmed these observations that the Lower@CSN and DLH@CSN/PGM groups had a higher proportion of EdU-positive cells compared to the Control and Upper@PGM groups (Figure 4D).

To evaluate the impact of the hydrogels on cellular migration, we performed cell scratch assay and transwell migration assay on NIH-3T3 cells. The cell scratch assay revealed that the cells treated with Lower@CSN and DLH@CSN/PGM had higher migration rates at 24 h (50.32% and 36.65%, respectively) and 48 h (79.39% and 71.32%, respectively), as evidenced by the reduced scratch width (Figure 4B). Conversely, the Upper@PGM group displayed the lowest migration rates at both time points (18.83% at 24 h and 53.29% at 48 h), which was consistent with the inhibitory effect of PFD. Moreover, there were no significant differences between the Control and DLH groups. Quantitative analysis of the scratch assay supported these findings (Figure 4E). The transwell migration assay further demonstrated the promoting effect of Lower@CSN and DLH@CSN/PGM on cell migration, with a significantly higher number of cells migrating through the membrane compared to the Control and Upper@PGM groups (Figure 4C). Quantitative analysis of the transwell assay confirmed these results, with the Lower@CSN and DLH@CSN/PGM groups showing a substantial increase in the number of migrating cells (Figure 4F). In a word, the DLH@CSN/PGM effectively promoted cell proliferation and migration during the early stages of wound healing through the release of Cur from the lower layer. The system also inhibited excessive proliferation in the later stages through the release of PFD from the upper layer, as evidenced by the reduced cell proliferation and migration in the Upper@PGM group. This sequential drug delivery mechanism aligns with the requirements for optimizing the wound healing process, potentially leading to rapid and scar-free recovery.

### 3.8. DLH@CSN/PGM Accelerates Wound Healing and Enhances Scar Quality

**Figure 5:**
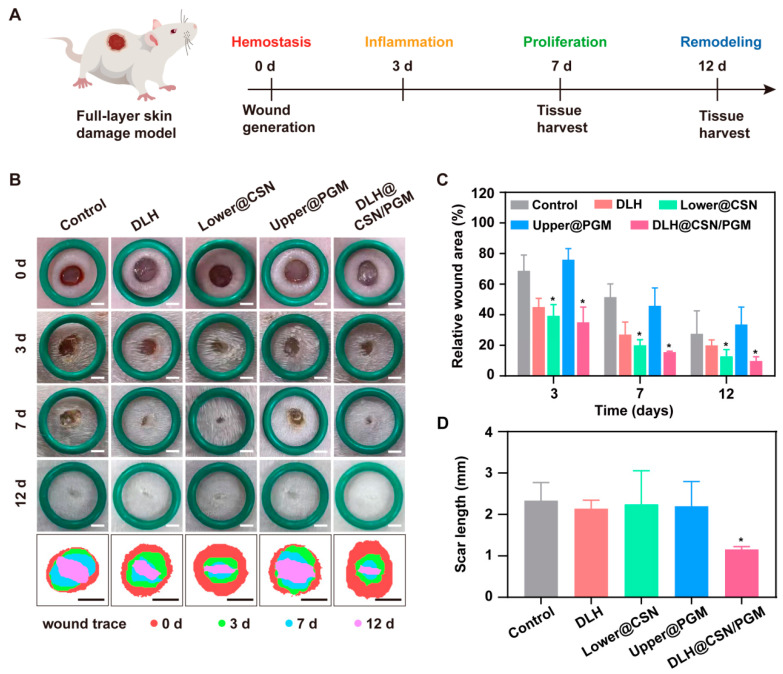
In vivo assessment of hydrogel effects on wound healing. (**A**) Schematic representation of the full-layer skin damage model and the timeline of the in vivo experiment. (**B**) Photographic documentation and simulated diagrams of wound sites at 0, 3, 7, and 12 days post-wounding, bar: 2 mm. (**C**) Quantitative analysis of relative wound area, * *p* < 0.05 vs. Control. (**D**) Quantification of scar length from the wound photographs, * *p* < 0.05 vs. Control.

The novel double-layer hydrogel system was designed to facilitate sequential drug release, addressing the distinct phases of wound healing. Combining Cur-loaded lower layers for early anti-inflammatory effects and PFD-encompassing upper layers for late-stage anti-fibrotic action was expected to optimize both the healing speed and the quality of the scar. To evaluate the efficacy of the DLH@CSN/PGM in wound healing, a rat model with a full-layer skin defect was established (Figure 5A). The wound healing process was assessed on 0, 3, 7, and 12 days post-wounding, with photographic documentation and computational simulation for visually monitoring the healing progression (Figure 5B). The images clearly showed that the Lower@CSN and DLH@CSN/PGM groups exhibited fast wound contraction, and the DLH@CSN/PGM group demonstrated the most significant improvement by day 12. Quantitative analysis of the relative wound area (Figure 5C) provided a more detailed view of the healing process. The DLH@CSN/PGM group showed significant reductions in wound area, with 35% and 15.6% remaining on days 3 and 7, respectively. This demonstrated the hydrogel’s potential to promote wound healing during the early and proliferative stages. In contrast, the Upper@PGM group, which contained PFD, showed relatively larger wound areas at each time point (76%, 45.8%, and 33.7%) due to the anti-proliferative effects of PFD. The scar length, a critical parameter for assessing the quality of wound healing, was also quantified (Figure 5D). The DLH@CSN/PGM group produced the shortest scar length at the end of the observation period. The result underscores the hydrogel’s ability to not only accelerate wound closure but also improve the quality of the scar.

### 3.9. DLH@CSN/PGM Modulates Oxidative Stress and Inflammation in Vivo

**Figure 6:**
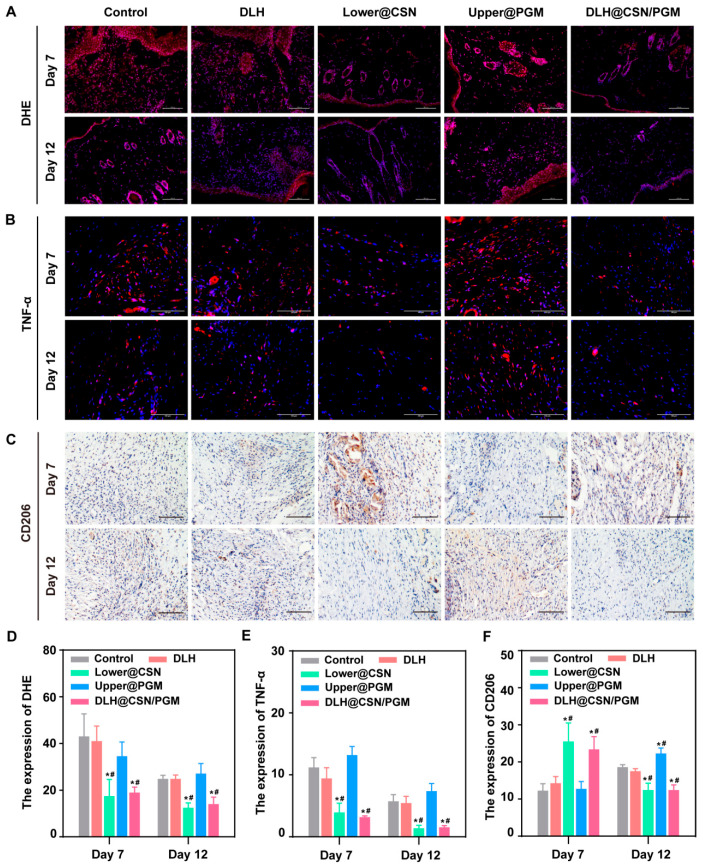
Assessment of DLH@CSN/PGM effects on oxidative stress and inflammation in wound healing. Immunofluorescence staining for (**A**) DHE and (**B**) TNF-α. Blue indicates the stained cell nuclei, and red indicates the stained protein expression, bar: 100 μm. (**C**) Immunohistochemical staining for CD206 of wounds on days 7 and 12, bar: 100 μm. (**D**) Quantitative analysis of DHE and (**E**) TNF-α fluorescence intensity. (**F**) Quantitative analysis of CD206 expression. * *p* < 0.05 vs. Control; # *p* < 0.05 vs. DLH.

Wound healing is a dynamic biological process involving inflammation, tissue formation, and remodeling. However, excessive inflammation and oxidative stress during the early phases can disrupt healing and lead to scar formation. ROS, which is produced by inflammatory cells, can control the infection at moderate levels and their excessive production will induce oxidative stress, apoptosis, and immune dysregulation [38]. Similarly, prolonged inflammation mediated by cytokines like tumor necrosis factor-α (TNF-α) hampers the healing process and exacerbates scar formation [39]. To evaluate the ability of DLH@CSN/PGM to regulate oxidative stress and inflammation, the ROS level, TNF-α expression, and macrophage polarization were assessed in vivo. DHE staining was used to quantify ROS production in wound tissues (Figure 6A). On day 7, the red fluorescence intensity indicating the ROS was significantly reduced in the Lower@CSN and DLH@CSN/PGM groups compared to the Control and Upper@PGM groups (Figure 6D). This reduction persisted through the experimental period, suggesting their sustained antioxidant effect. Conversely, the Upper@PGM group displayed much higher fluorescence intensity at both time points compared with these two groups. The reduction in ROS levels in the DLH@CSN/PGM group could be attributed to the controlled release of Cur in the lower hydrogel layer, emphasizing its antioxidant properties during the inflammatory phase.

TNF-α, a hallmark pro-inflammatory cytokine, was evaluated through immunofluorescence staining (Figure 6B). The red fluorescence intensity representing TNF-α was significantly lower in the Lower@CSN and DLH@CSN/PGM groups on days 7 and 12 (Figure 6E). In contrast, the Control and Upper@PGM groups exhibited persistently higher levels of TNF-α, reflecting prolonged inflammation. The reduced TNF-α level in the DLH@CSN/PGM group suggests an effective inflammation modulation, facilitating a balanced immune response that supports wound resolution without chronic inflammation.

Macrophages are critical mediators of wound repair, with M2 macrophages exhibiting anti-inflammatory and tissue-promoting roles. However, excessive M2 macrophage activity during the remodeling phase can lead to hypertrophic scarring due to excessive collagen deposition [40]. CD206, a marker of M2 macrophages, was evaluated through immunohistochemistry (Figure 6C). On day 7, both the Lower@CSN and DLH@CSN/PGM groups showed higher green fluorescence intensity, indicating enhanced M2 macrophage polarization, which aids in resolving inflammation and promoting tissue regeneration. On day 12, CD206 expression in these groups significantly decreased (Figure 6F), demonstrating timely regulation of M2 macrophage activity. In contrast, other groups displayed the opposite trend, with persistently high or increasing CD206 levels on day 12, potentially leading to excessive collagen deposition and poor scar quality. The DLH@CSN/PGM’s ability to modulate M2 polarization may be derived from the sequential release of Cur and PFD, ensuring inflammation resolution without promoting fibrosis.

### 3.10. In Vivo Angiogenesis Enhancement by DLH@CSN/PGM

**Figure 7:**
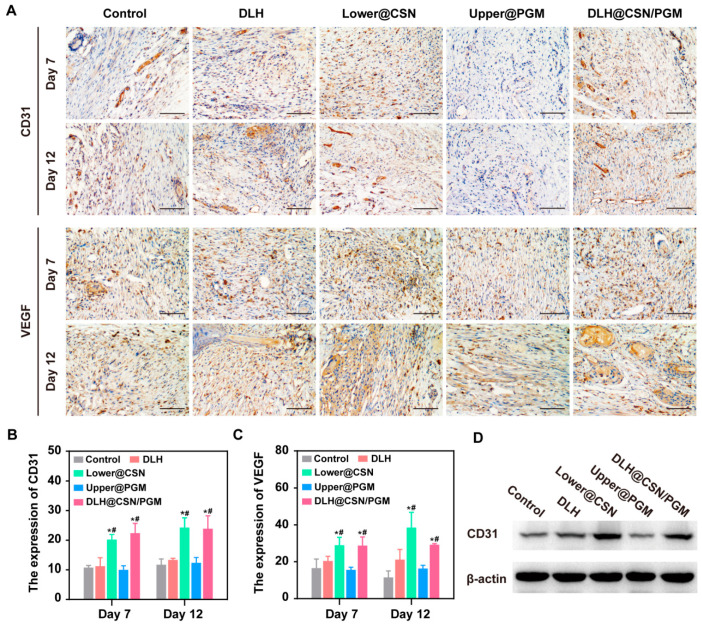
In vivo assessment of DLH@CSN/PGM-mediated enhancement of wound angiogenesis. (**A**) Immunohistochemical staining for CD31 and VEGF in rat skin wound tissue on days 7 and 12, bar: 100 μm. (**B**,**C**) Quantitative analysis of CD31 and VEGF expression of immunohistochemical staining, * *p* < 0.05 vs. Control; # *p* < 0.05 vs. DLH. (**D**) Western blot analysis of CD31 protein expression in wound tissue on day 7.

Following the initial inflammatory phase of wound healing, the formation of new blood vessels during the proliferative phase is essential for effective tissue regeneration. However, uncontrolled angiogenesis during the remodeling phase can lead to the development of non-functional blood vessels and exacerbate scar formation [41]. To evaluate the impact of DLH@CSN/PGM on angiogenesis in vivo, IHC and Western blot analyses were employed using a rat skin wound model. IHC staining for CD31 (a marker of endothelial cells) and VEGF (a key mediator of neovascularization) was performed on rat skin wound tissue on days 7 and 12 post-wounding (Figure 7A). Quantitative analysis of CD31 and VEGF expression revealed a significant increase in the Lower@CSN and DLH@CSN/PGM groups compared to the Control and DLH groups, indicating enhanced angiogenesis (Figure 7B,C). The Western blot analysis of CD31 protein expression on day 7 further confirmed the robust angiogenic response with up-regulation of CD31 in the Lower@CSN and DLH@CSN/PGM groups (Figure 7D and Figure S9). The results suggested that the sequential drug release from the DLH@CSN/PGM hydrogel system promoted rapid angiogenesis during the early proliferative phase while potentially preventing excessive angiogenesis in the later remodeling phase.

### 3.11. DLH@CSN/PGM Promotes Reepithelialization and Collagen Deposition

**Figure 8:**
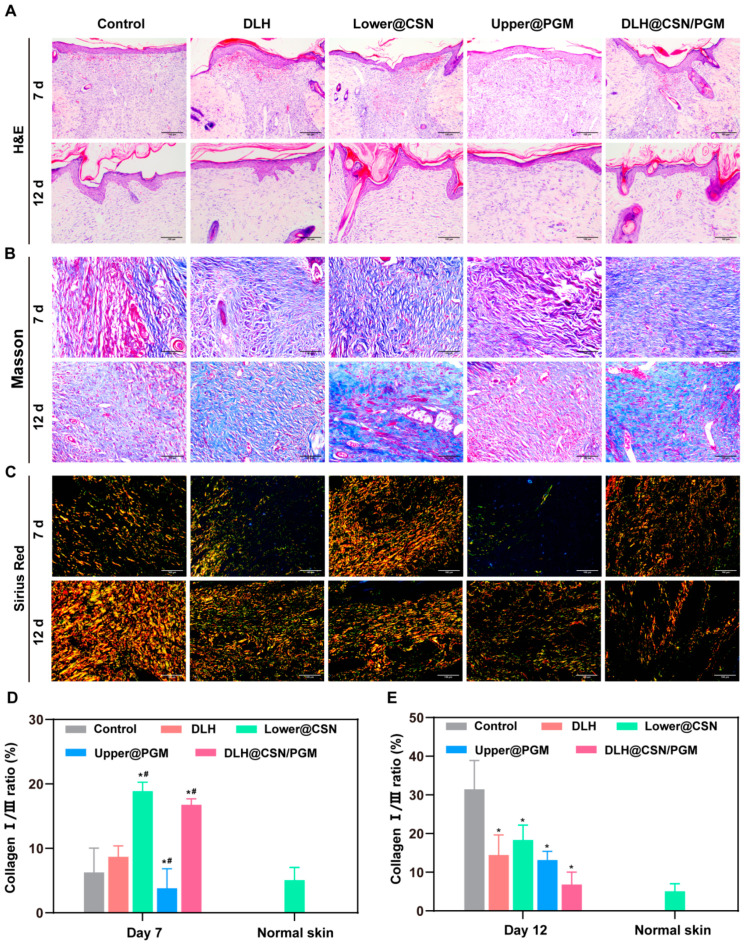
In vivo analysis of hydrogel impact on wound healing and collagen deposition. (**A**) H&E staining, (**B**) Masson staining, and (**C**) Sirius Red staining of wound tissue sections on days 7 and 12, bar: 100 μm. (**D**,**E**) The quantitative analysis of the collagen I/III ratio on days 7 and 12, * *p* < 0.05 vs. Control; # *p* < 0.05 vs. DLH.

To further evaluate the therapeutic efficacy of DLH@CSN/PGM on wound healing, histological analysis was conducted to assess reepithelialization and collagen deposition during the healing process. Granulation tissue formation, collagen synthesis, and the epithelialization process were monitored [42], with a particular focus on the transition from early proliferation to the remodeling phase. H&E staining results revealed significant differences in tissue regeneration between the treatment groups. On day 7, the DLH@CSN/PGM and Lower@CSN groups demonstrated extensive granulation tissue proliferation, characterized by an increased presence of fibroblasts, neovascularization, and inflammatory cells. On day 12, the DLH@CSN/PGM-treated wounds exhibited a highly organized and complete epithelial structure, indicating advanced reepithelialization. In contrast, the Control and Upper@PGM groups showed incomplete epithelialization with residual visible defects (Figure 8A). These findings suggest that the double-layer hydrogel system fosters rapid and effective reepithelialization, which is essential for wound closure.

Collagen, a key component of the ECM, provides structural support for wound healing. The Masson staining results revealed a marked increase in collagen deposition in the Lower@CSN and DLH@CSN/PGM groups on day 12, with a more orderly collagen arrangement in the DLH@CSN/PGM group compared to the chaotic collagen organization observed in the Control group (Figure 8B). These results indicated that DLH@CSN/PGM not only accelerated collagen production but also enhanced its structural arrangement, contributing to the strength and quality of the healed tissue.

Sirius Red staining was employed to distinguish between Type I and Type III collagen, the primary ECM components during wound healing. Immature Type III collagen predominates during the early proliferative phase and is gradually replaced by more elastic Type I collagen as healing progresses [43]. On day 7, both the Lower@CSN and DLH@CSN/PGM groups exhibited a significant increase in Type I collagen deposition compared to other groups (Figure 8D). On day 12, the DLH@CSN/PGM group showed a Type I/III collagen ratio close to normal skin (Figure 8C,E and Figure S8), underscoring its ability to balance ECM remodeling and scarring. The fibrotic inhibitory effect of PFD, released from the upper hydrogel layer in the later stages of healing, contributed to this favorable outcome. The results of histological evaluations substantiate the efficacy of the DLH@CSN/PGM hydrogel in promoting high-quality wound healing. By facilitating early granulation tissue formation and precise regulation of collagen deposition, the double-layer hydrogel system supports the structural and functional restoration of the wound, achieving outcomes close to normal skin. These findings validate the strategic design of DLH@CSN/PGM for phase-specific drug release and underscore its potential for advancing scar-free wound management therapies.

## 4. Conclusions

In summary, this study presented a DLH@CSN/PGM that addresses the multifaceted challenges of scar-free wound healing by providing sequential drug release tailored to the wound healing process. The DLH@CSN/PGM demonstrated excellent biocompatibility and mechanical stability, which was essential for effective wound dressing applications. The Lower@CSN layer, with its rapid release of Cur, effectively mitigated inflammation and oxidative stress during the early stages, while the upper@PGM layer, with its sustained release of PFD, inhibited excessive fibrosis and collagen deposition in the later stages. In vivo experiments confirmed the ability of DLH@CSN/PGM to enhance early granulation tissue formation, promote wound closure, and improve scar quality without compromising hair follicle regeneration. These findings underscore the potential of DLH@CSN/PGM in optimizing the wound healing process, preventing pathological scarring, and facilitating the restoration of healthy skin.

## Data Availability

The raw data supporting the conclusions of this article will be made available by the authors on request.

## Supplementary Materials

The following supporting information can be downloaded at https://www.mdpi.com/article/10.3390/jfb16050164/s1, Figure S1. FTIR spectra of the chitosan (Cs), curcumin (Cur), chitosan nanoparticles (CNPs) and curcumin-loaded chitosan nanoparticles (CSNs); Figure S2. FTIR spectra of the gelatin (Gel), pirfenidone (PFD), gelatin microspheres (GEMs), and pirfenidone-loaded gelatin microspheres (PGMs); Figure S3. TEM images of CNPs and CSNs; Figure S4. SEM images of CSNs, GEMs, and PGMs; Figure S5. The swelling ratios of the upper layer and lower layer; Figure S6. Human umbilical vein endothelial cells (HUVECs) viability assessed after 24 and 48 h of treatment with various hydrogel extracts; Figure S7. In vivo photographs illustrating the liver bleeding model and the quantitative analysis; Figure S8. The normal skin’s representative images of Sirius Red staining; Figure S9. Western blot analysis of CD31 protein expression levels in rat skin wound tissue on day 7 post-treatment; Figure S10. The calibration curves of Cur; Figure S11. The calibration curves of PFD; Figure S12. Photographs of DLH@CSN/PGM.
