# Supplementary material for: Biodegradable Double-Layer Hydrogels with Sequential Drug Release for Multi-Phase Collaborative Regulation in Scar-Free Wound Healing
Source: J Funct Biomater. 2025 May 7;16(5):164. doi: 10.3390/jfb16050164 (PMC12111865; doi:10.3390/jfb16050164)
Supplement: Supplementary file 1 [file jfb-16-00164-s001.zip › jfb-3559858-supplementary.pdf]

Journal of
Functional Biomaterials

MDPI

Article

Biodegradable Double-Layer Hydrogels with Sequential Drug Release for Multi-Phase Collaborative Regulation in Scar-Free Wound Healing

Xinyu Zhang [1], Qianhe Zu[2], Chunlin Deng [3], Xin Gao [3], Hongxu Liu [1], Yi Jin [2], Xinjian Yang [3,*] and Enjun Wang [1,*]

[1] College of Nursing, Hebei University, Baoding 071002, PR China
[2] College of Basic Medical Science, Key Laboratory of Pathogenesis Mechanism and Control of Inflammatory-autoimmune Diseases of Hebei Province, Hebei University, Baoding 071002, P. R. China
[3] College of Chemistry & Materials Science, State Key Laboratory of New Pharmaceutical Preparations and Excipients, Key Laboratory of Medicinal Chemistry and Molecular Diagnosis of the Ministry of Education, Chemical Biology Key Laboratory of Hebei Province, Institute of Life Science and Green Development, Hebei University, Baoding 071002, P. R. China
* Correspondence: xjyang321@hbu.edu.cn; wej2009@hbu.edu.cn

Supplementary experimental section

Materials

Sodium alginate (SA), cysteine methyl ester hydrochloride (CME·HCl), 1-(3-Dimethylaminopropyl)-3-ethylcarbodiimide (EDC), N-Hydroxysuccinimide (NHS), polyethylene glycol diacrylate (PEG-DA), sodium tripolyphosphate (TPP), Gelatin (Gel), pirfenidone (PFD), photoinitiator I2959 and Span-80 were purchased from Aladdin Biochemical Technology Co., Ltd. Curcumin (Cur) and chitosan (Cs) were purchased from Ron Reagent Co., Ltd. Phosphate buffered saline (PBS), Dulbecco's modified Eagle medium (DMEM) and fetal bovine serum (FBS) were purchased from Dalian Meilun Biotechnology Co., Ltd.

Preparation and characterization of thiolated alginate (SA-SH)

Firstly, the 3 g of EDC and 3 g of NHS were dissolved in 100 mL of 2% sodium alginate solution (pH: 6.0) to react for 1 h. The pH of the solution was adjusted to 7.4, and then, 4 g of CME·HCl was added to the solution for a reaction of 24 h. Finally, the materials were purified and washed with ethanol and ether, respectively. SA-SH was obtained after vacuum drying the material for 24 h.

Preparation and characterization of curcumin-loaded chitosan nanoparticles (CSN)

CSN were prepared using an ionic gelation method. A total of 6.67 mg of Cur was dissolved in 10 mL of a 0.2% chitosan aqueous solution (prepared in 1% v/v acetic acid, pH 5.0). Nanoparticles were spontaneously formed by the dropwise addition of a sodium tripolyphosphate (TPP) aqueous solution to the mixed solution under continuous stirring at 30°C for 60 minutes. The resulting suspension was centrifuged at 12,000 rpm and 4°C for 30 minutes. The sedimented nanoparticles were washed with deionized water and subsequently lyophilized for storage. The morphology of the CSN was examined using transmission electron microscopy (TEM) (Tecnai G2 F20 STwin). Functional group analysis of the fabricated scaffolds was performed using Fourier Transform Infrared Spectroscopy (FTIR) (Nicolet iS10). Spectral measurements were conducted at a resolution of 4 cm^{-1} over a frequency range of 4000–400 cm^{-1}.

Preparation and characterization of pirfenidone-encapsulated gelatin microspheres (PGM)

PGM was prepared using an emulsion crosslinking method. For the aqueous phase, 1.1 g of gelatin was dissolved in 45 mL of deionized water and stirred at 60°C for 1 hour. The oil phase was prepared by adding 0.7 mL of Span-80 to 22.2 mL of liquid paraffin and stirring at 50°C for 10 min. Subsequently, 10 mL of the aqueous phase was injected dropwise into the oil phase under continuous agitation at 50°C for 10 min. The mixture was rapidly cooled to below 10°C by immersion in an ice-water bath for 15 min. To initiate crosslinking, 0.5 mL of a 50% (w/v) glutaraldehyde solution was added, and the mixture was stirred at a low temperature for 60 min to ensure complete crosslinking. The microspheres were then washed thoroughly to remove residual oil and dried under vacuum for 24 h. The dried gelatin microspheres (GEM) were subsequently loaded with PFD by stirring in an aqueous PFD solution for 4 h. The loaded microspheres were then centrifuged, washed, and vacuum-dried for 24 h. The morphology and grain size of the microspheres were analyzed using scanning electron microscopy (SEM) (Nova NanoSEM 450). Func-

tional group characterization of the fabricated scaffolds was performed using FTIR. Spectral measurements were obtained at a resolution of 4 cm^{-1} over the frequency range of 4000~400 cm^{-1}.

Characterization:

For SEM analysis: The tested sample was placed in liquid nitrogen for 10 min and freeze-dried for 24 h. The dried sample was mounted on a conductive adhesive surface and sputter-coated with a thin layer of gold to enhance electrical conductivity. The coated sample was then analyzed using a SEM to observe morphological features at various magnifications.

For TEM: An aqueous suspension of CSN was prepared at an appropriate concentration and sonicated to ensure homogeneity. A volume of 200 μL of the suspension was carefully deposited onto a copper grid and allowed to air-dry. The grid was then examined using a TEM to visualize the nanoparticle morphology and structure.

For FTIR analysis: A mixture of potassium bromide and the sample powder (in a ratio of 100:1) was finely ground and pressed into a transparent pellet. The pellet was analyzed using an FTIR to obtain spectral data, with measurements taken at the thinnest point of the pellet to ensure optimal spectral resolution.

For Raman analysis: The sample was placed on the sample pad in a dark environment to minimize background interference. The sample was then scanned to collect Raman spectral data.

Detection of drug loading rate (CSN and PGM)

CSN: 0.5 mg of CSN was weighted and 30 mL of absolute ethanol as the extract of Cur was added. The mixture was centrifuged at 10000 rpm for 10 min. Then, the concentration and weight of Cur in the supernatant liquid were tested by detecting the absorbance at 425 nm using UV-visible spectroscopy and employing a pre-tested standard curve using UV-visible spectroscopy. The drug loading rate is calculated by the following equation.

$$\text{Drug loading rate (\%)} = (m_2/m_1) \times 100 \quad (1)$$

Where m_1 is the weight of CSN and m_2 is the weight of Cur in the supernatant liquid.

PGM: 1 mg of GEM was added to 0.06 mg/mL of PFD solution (PBS) to stir for 4 h. The mixture was centrifuged at 8000 rpm for 10 min, and then, the absorbance of the supernatant at 281 nm was detected by UV-visible spectroscopy. The concentration and weight of PFD in the supernatant liquid were calculated by using a pre-tested standard curve. The drug loading rate are calculated by the following equations.

$$\text{Drug loading rate (\%)} = [(m_2-m_3)/(m_1+m_2-m_3)] \times 100 \quad (2)$$

Where m_1 is the weight of GEM, m_2 is the weight of dosage of PFD, and m_3 is the weight of free-floating drugs.

Preparation of dual-layer hydrogels (DLH)

DLH was synthesized following a previously reported method with minor modifications. Briefly, 10 mg of the photoinitiator I2959 was dissolved in 5 mL of deionized water, and a specific amount of polyethylene glycol diacrylate (PEG-DA) was added to the solution. The lower layer of the DLH was prepared using 100 μL of PEG-DA (Mn=400), while the upper layer consisted of 165 μL of PEG-DA (Mn=700). The resulting solution was added to a sample vial containing 0.075 g of SA-SH powder and stirred rapidly for 10

minutes. The sample was then irradiated under a UV lamp at 365 nm. Following UV exposure, the DLH was removed and placed in a dialysis bag (molecular weight cutoff of 14 kDa) for purification by dialysis against deionized water for 24 h at room temperature, which eliminated any unreacted I2959 and PEG-DA.

$$S_{eq}\,(\%) = \frac{W_{eq} - W_d}{W_d} \times 100 \text{Remaining hydrogel (\%)} = \frac{W_t}{W_0} \times 100 \quad (3)$$

Swelling experiment

The swelling behavior of the upper and lower hydrogel layers was evaluated by immersing the samples in phosphate-buffered saline (PBS, pH = 7.4) at 37°C. After a swelling period of 15 min, excess surface liquid was removed, and the swollen hydrogels were weighed. The swelling rate (S_{eq}%) was determined using the following equation:

$$S_{eq}\,(\%) = \frac{W_{eq} - W_d}{W_d} \times 100 \quad (4)$$

Where W_{eq} represents the mass of the swollen hydrogel, and W_d represents the mass of the dry hydrogel.

Rheological experiment

The rheological properties of the hydrogels were assessed using a rotational rheometer (Mars40, Thermo Fisher Scientific). Frequency sweep measurements were conducted by varying the oscillation frequency from 0.01 Hz to 10 Hz at a fixed strain of 1 %. All tests were performed at a physiological temperature of 37°C.

Degradation test

The degradation behavior of the hydrogels was analyzed by immersing samples in PBS to mimic their degradation in physiological fluids. At predefined time intervals, the hydrogels were removed, freeze-dried, and weighed. The remaining mass of the hydrogels was calculated using the following equation:

$$\text{Remaining hydrogel (\%)} = \frac{W_t}{W_0} \times 100 \quad (5)$$

where W_t and W_0 are the weights of the hydrogel at time *t* and the initial dry weight, respectively.

In vitro biocompatibility

Hydrogel extracts were prepared by incubating the different hydrogels in PBS for 24 h at a concentration of 0.05 mg/mL. The supernatant was then collected for further analysis. The biocompatibility of the hydrogels was evaluated using a CCK-8 assay to assess HUVEC proliferation at days 1 and 2, following the manufacturer's protocol. Optical density (OD) values at 450 nm were measured using a multifunctional microplate reader (BX-53). A Live/Dead assay was conducted to determine cell viability by incubating NIH/3T3 cells (2×10^4 cells/well) with 500 μL of hydrogel extract for 24 h. After staining with calcein-AM/propidium iodide, cells were imaged using an inverted fluorescence microscope. NIH/3T3 cell morphology was analyzed using rhodamine-phalloidin/DAPI staining.

The blood compatibility of the hydrogels was assessed via a hemolysis test. Red blood cells from rat blood were incubated with 100 μL hydrogel extract, PBS (negative

control), or water (positive control). Hemolysis was quantified by measuring the absorbance of supernatants at 540 nm.

Hemostatic ability

The hemostatic ability of DLH@CSN/PGM was evaluated using an in vivo liver hemorrhage model. Briefly, rats were anesthetized, and a laparotomy was performed to expose the liver. A surgical blade was used to induce liver injury, and DLH@CSN/PGM was applied topically to the wound site. Hemostatic efficacy was determined by measuring the bleeding weight.

Angiogenesis study

The angiogenesis in the wound was assessed via immunohistochemical staining of CD31 and VEGF. Rat skin paraffin sections (4-5 μm) were deparaffinized and underwent citrate-based antigen retrieval at 95 °C for 20 min. After blocking with 5% BSA, sections were incubated overnight at 4 °C with anti-CD31 (1:100, Abcam ab56299) or anti-VEGF (1:200, Santa Cruz sc-152). They were then treated with HRP-conjugated secondary antibodies (1:500) and developed with DAB. Sections were counterstained with hematoxylin, and CD31-positive vessels or VEGF-positive areas were quantified using Image J.

Supplementary figure captions

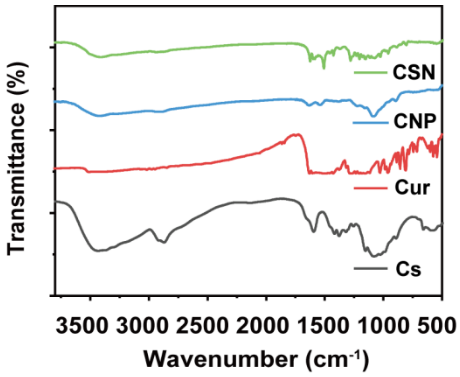

Figure S1. FTIR spectra of the chitosan (Cs), curcumin (Cur), chitosan nanoparticles (CNP) and curcumin-loaded chitosan nanoparticles (CSN).

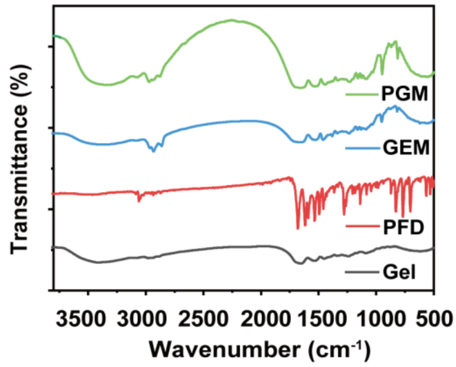

Figure S2. FTIR spectra of the gelatin (Gel), pirfenidone (PFD), gelatin microspheres (GEM), and pirfenidone-loaded gelatin microspheres (PGM).

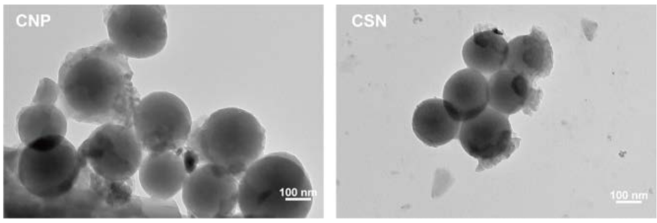

Figure S3. TEM images of CNP and CSN.

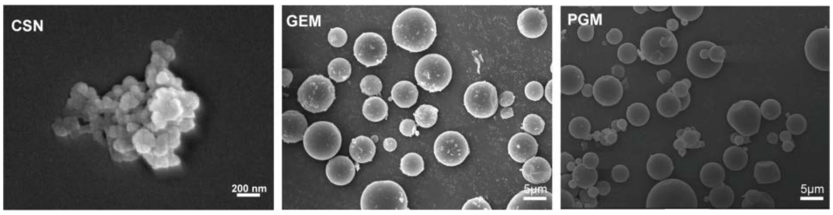

Figure S4. SEM images of CSN, GEM and PGM.

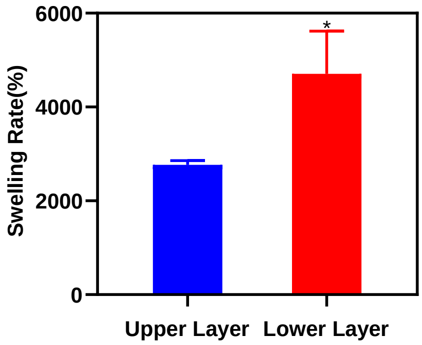

Figure S5. The swelling ratios of Upper Layer and Lower Layer.

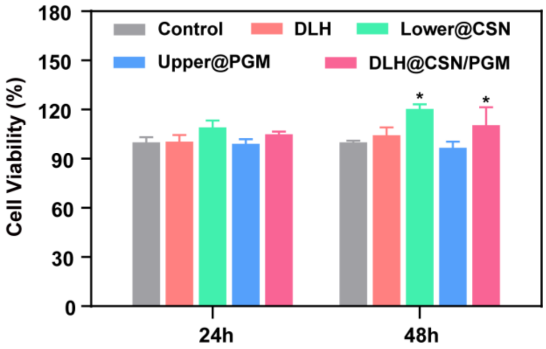

Figure S6. Human Umbilical Vein Endothelial Cells (HUVECs) viability assessed after 24 and 48 h of treatment with various hydrogel extracts. *$p < 0.05$ vs Control.

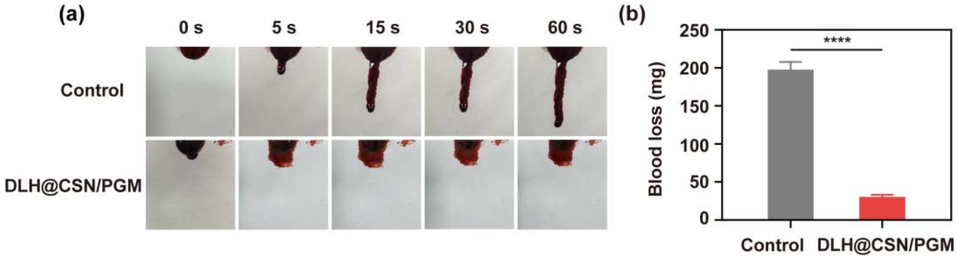

Figure S7. (a) In vivo photographs illustrating the liver bleeding model at 0, 5, 15, 30, and 60 s for both Control and DLH@CSN/PGM treated groups. (b) Quantitative analysis of blood loss for the Control and DLH@CSN/PGM treated groups. $^{****}p < 0.0001$ vs Control.

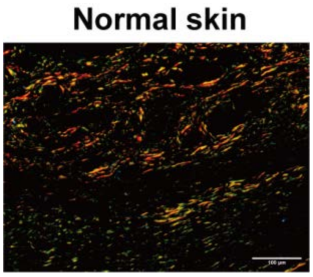

Figure S8. The normal skin's representative images of sirius red staining, bar: 100μm.

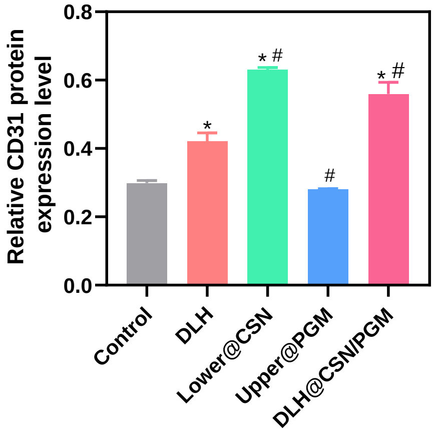

Figure S9. Western blot analysis of CD31 protein expression levels in rat skin wound tissue on day 7 post-treatment. $^{*}P < 0.05$ vs Control; $^{\#}P < 0.05$ vs DLH.

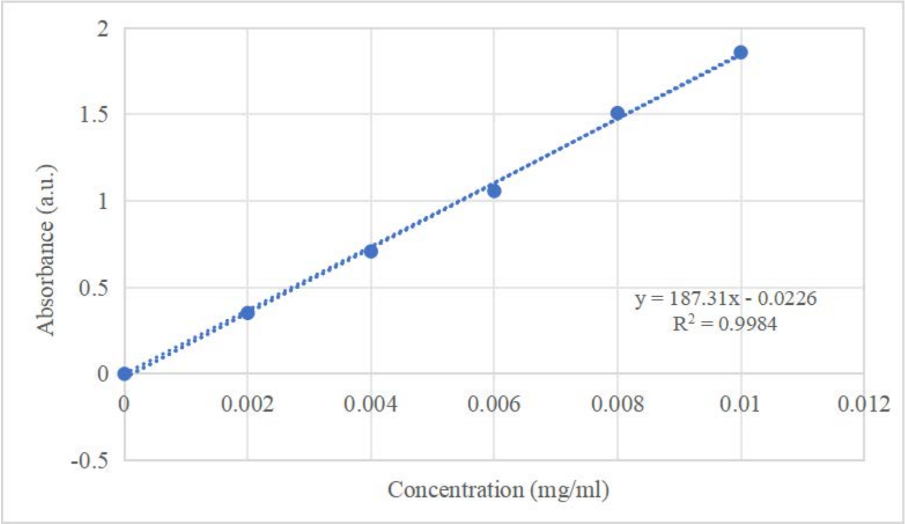

Figure S10. The calibration curves of Cur.

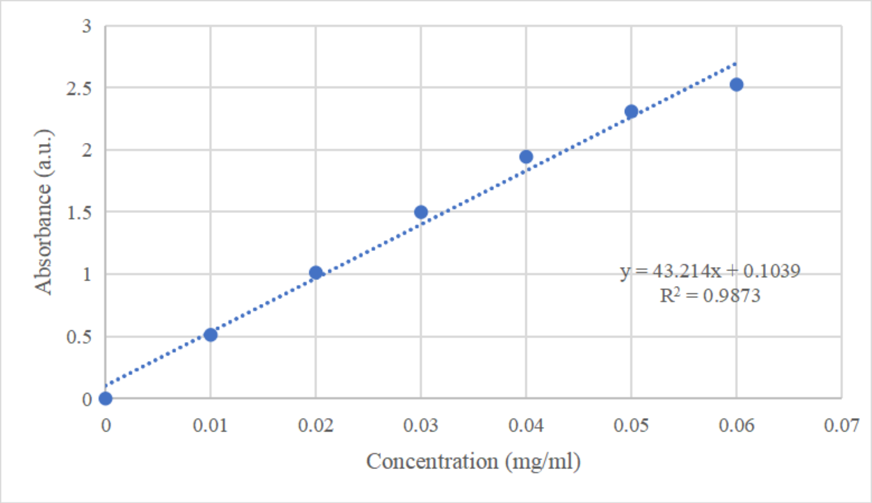

Figure S11. The calibration curves of PFD.

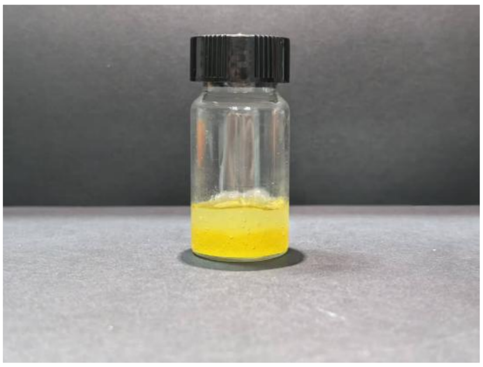

Figure S12. Photographs of DLH@CSN/PGM.
